# The Frst Physician in Msssachusetts

**Published:** 1869-06

**Authors:** 


The First Physician in Massachusetts.—Dr. Samuel Fuller, the physician of the Mayflower, was the first disciple of Galen mentioned in the history of Massachusetts.

